# The Influence of Soft Segment Structure on the Properties of Polyurethanes

**DOI:** 10.3390/polym15183755

**Published:** 2023-09-14

**Authors:** Ivan Ristić, Suzana Cakić, Nevena Vukić, Vesna Teofilović, Jelena Tanasić, Branka Pilić

**Affiliations:** 1 Faculty of Technology Novi Sad, University of Novi Sad, Bulevar cara Lazara 1, 21102 Novi Sad, Serbia; vesnateofilovic@uns.ac.rs (V.T.); jelenatanasic@uns.ac.rs (J.T.); brapi@uns.ac.rs (B.P.); 2 Faculty of Technology, University of Niš, Bulevar Oslobođenja 124, 16000 Leskovac, Serbia; cakics@tf.ni.ac.rs; 3 Faculty of Technical Sciences, University of Kragujevac, Svetog Save 65, 32102 Čačak, Serbia

**Keywords:** polyurethane, poly(lactide), polyester polyols, thermal analysis, phase separation

## Abstract

A series of polyurethanes (PU) were synthesised via one-step polymerisation without a chain extender, using toluene diisocyanate as well as a variety of soft segments composed of different macrodiols. Poly(D,L-lactide) (PDLLA) and polycaprolactone diol (PCL) were synthesised as a polyester type polyols to obtain soft segments. The process of varying the molar ratio of newly synthesised PDLLA in soft segments has been confirmed as a powerful tool for fine-tuning the final properties of PU. Fourier-transformed infrared spectroscopy was used for evaluation of molecular structures of synthesised PDLLA polyol and final PU. Nuclear magnetic resonance spectrometry was used to confirm the presumed structure of PU. The influence of soft segment composition on polyurethane thermal characteristics was examined using thermogravimetric analysis and differential scanning calorimetry. The composition of soft segments had little impact on the thermal stability of PU materials, which is explained by the comparable structures of both polyester polyols. Wide-angle X-ray scattering was utilised to evaluate the effect of amorphous PDLLA on the degree of crystallinity of PCL in soft PU segments. It was discovered that not only did the PDLLA ratio in the soft segment have a substantial influence on the degree of microphase separation in the soft and hard segments, but it also influenced the crystallisation behaviour of the materials. Furthermore, the restriction of crystallisation of the PCL soft segment has been verified to be dependent on the hard segment concentration and the ratio of PDLLA/PCL polyols. The sample with pure PCL as the polyol component achieved the highest degree of crystallinity (34.8%). The results demonstrated that the composition of soft segments directly affected the properties of obtained polyurethane films. These results can be utilised to easily achieve a desirable set of properties required for application in biomaterials.

## 1. Introduction

The versatility of polyurethane (PU) chemistry enables the synthesis of novel polyurethanes based on renewable raw materials with a broad range of chemical and physical properties [1]. The synthesis of biocompatible polyurethane is of immense interest in the biomedical and pharmaceutical fields [2]. The properties of these materials must be fine-tuned in order to prevent negative effects on the surrounding tissue [3]. Polyurethanes find application in various long-term implantable devices, such as scaffolds, cardiac valves, and ocular implants [4], as well as in commercial products intended for removal after use, such as wound dressings [5,6]. For all mentioned applications, especially when a certain level of elasticity is required, segmented polyurethanes, consisting of soft and hard segments, are commonly chosen materials [7]. The soft segment is an alternating flexible component or macrodiol, whereas the hard segment is a stiff component generated from diisocyanate and a chain extender. The segment structure, hard/soft ratio, molecular weight of polyols, and the type of chain extender, if used, all determine the PU’s properties.

To increase the sustainability of polyurethane materials, various strategies have been explored, including the development of non-isocyanate PUs and the use of plastic waste or biomass as carbon sources [8]. Among the renewable and biodegradable materials, poly(lactide) (PLA) has garnered significant attention in polyurethane chemistry. The monomer used to make this polyester polyol is primarily obtained from starch and sugar [9]. It can be hydrolysed into lactic acid, making it metaboliseable in vivo and environmentally friendly, thus offering a wide range of applications [10]. PLA serves as a sustainable alternative to petrochemical polyols. Being a biodegradable material, poly(D,L-lactide) (PDLLA) may be tailored to deteriorate over time, making it highly suitable for a variety of biomedical and environmental applications [11].

Due to their excellent mechanical properties, commercially available biodegradable polyurethanes are often based on polyesters. They are, however, susceptible to hydrolytic destruction when implanted in the body, even for brief periods. To address this, polycaprolactone [12], polylactide [13], vegetable oils [14,15,16,17], and other biobased polyols [18,19,20] have been utilised. 

In polyurethanes, the inclusion of polycaprolactone diol (PCL) typically enhances crystallinity, as PCL is a semi-crystalline, biodegradable polymer with ester bonds susceptible to hydrolysis [21]. Various studies have explored PCL-based PUs containing urethane and urea groups to improve its mechanical properties for cardiac tissue engineering [12]. Waterborne biodegradable polyurethanes have been developed using different quantities of PCL and poly(ethylene glycol) (PEG) as the soft segment [22]. Additionally, biodegradable polyurethanes were synthesised with varying molar ratios and molecular weights of PCL and PEG as soft segment and 1,6-hexamethylenediisocyante (HMDI) as hard segment via one-step bulk polycondensation, and the mechanisms of degradation and mechanical properties of these polyurethanes were investigated [12]. Generally, an increase in the length of the polyol resulted in a decrease in the ratio of the hard segment, leading to an increased tendency of the polyurethanes to crystallise. In order to obtain shape memory materials, a set of polylactide polyurethanes (PLAPUs) was synthesised using poly(L-lactide) diols, hexamethylene diisocyanate (HDI), and 1,4-butanediol [23]. A PLA-based thermoplastic polyurethane was also prepared for 3D printing, utilising modified PLA-diol, dicyclohexylmethane-4,4′-diisocyanate, and 1,4-butanediol [24].

This study follows the preparation of a series of polyurethanes through the one-step polymerisation process. A novel approach was adopted to synthesise new polyol components based on poly(D,L-lactide) from different biorenewable resources for PU preparation. Additionally, the study examines materials formed by reacting toluene diisocyanate (TDI) as the hard segment and with varying ratios of PDLLA and PCL as the soft segment. The main focus of this research is influence of the soft segment composition on PU properties, which contributes to a better understanding of these materials and their potential applications in the field of biomaterials.

## 2. Materials and Methods

### 2.1. Materials

*(3S)-cis-*3,6-dimethyl-1,4-dioxane-2,5-dione (D,L-Lactide) (98% purity) (Sigma-Aldrich, Waltham, MA, USA); 1,4:3,6-dianhydro-D-glucitol (isosorbide) (98% purity) (Sigma-Aldrich), catalyst trifluoromethanesulfonic acid (99% purity, 1.696 g cm^−3^ density at 25 °C) (Sigma-Aldrich). Polycaprolactone diol (PCL) (1250 g mol^−1^ molecular weight, toluene diisocyanate (TDI, 98 wt% purity) (Sigma Aldrich). All other chemicals were of reagent grade and utilised as-is without additional purification.

### 2.2. Polymerisation of Poly(D,L-lacide) Polyol

In order to obtain poly(D,L-lactide) polyol with the desired molecular weight, isosorbide was used as a chain length regulator [25]. Cationic solution polymerisations were conducted in a three-necked glass reactor equipped with a magnetic stirrer, using dichloromethane as the solvent. The monomers, consisting of 27.8 g D,L-lactide and 1.7 g isosorbide, were introduced into the reactor under a nitrogen atmosphere. Subsequently, 70 cm^3^ of solvent was added to dissolve the monomers. To initiate the reaction, 0.7 cm^3^ of trifluoromethanesulfonic acid was added to the mixture, which was then stirred at room temperature for 1 h. Following this, the temperature was raised to 40 °C, and the polymerisation process was carried out for 4 h under a nitrogen atmosphere. The average molecular mass of the synthesised PDLLA was 1100 g mol^−1^, with polydispersity index 1,2, which is in accordance with our previous research, which showed that this type of polymerisation yields a polymer with a narrow distribution of molecular masses [25].

### 2.3. Synthesis of Polyurethanes

**Figure 1:**
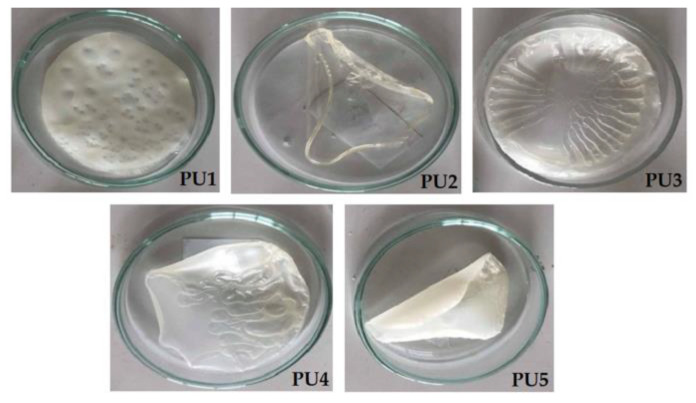
Prepared samples of polyurethane (PU).

The polyurethanes were prepared via a one-step solution polymerisation process. PCL and the necessary amount of PDLLA polyol were combined in tetrahydrofuran at 70 °C for 30 min to obtain a uniform mixture. All the samples were prepared with 25 wt% of the hard segment content. TDI and 1% stannous octoate catalyst were added, and the mixture was stirred for 2.5 h at 65 °C. Films were created by casting the PU solution in THF onto a glass surface and then left to dry at room temperature for two days. The obtained samples are shown in Figure 1. Table 1 shows the molar compositions of the raw materials used for the different polyurethanes (PU).

### 2.4. Characterisation of Obtained PU Samples

Gel permeation chromatography (GPC) was used to obtain the molecular weights and polydispersity index values of the polymers by using the GPC Waters system with pump 510 and 410 differential refractometer. Tetrahydrofuran (THF) with a flow rate of 1.0 mL min^−1^ at 30 °C was used as the eluent. Phenogel columns were used (four columns 300 × 7.8 mm ID; pore size 50; 100; 103 i 104 A, respectively). Polystyrene (PS) was used as the standard. Analyses were performed on a Waters GPC device (Milford, MA, USA) equipped with detectors: a ViscoStar type viscometer with a dilution factor of 0.5234; a DAWN EOS type light-scattering measuring device, with a K5 cell, the wavelength of the used laser set at 684.0 nm and a calibration constant of 1.0380 × 10^−5^ 1/V cm; and an RI detector-type Optiolab rEX. The analyses were conducted using chloroform as the eluent at a flow rate of 1 mL min^−1^.

Infrared spectra were obtained using a Fourier transform infrared spectrophotometer (FTIR) IRAffinity-1S (Shimadzu, Tokyo, Japan). Measurements were carried out using ATR (aAttenuated otal reflection) in the range 4000–400 cm^−1^, with a resolution of 4 cm^−1^.

^1^H NMR analysis of synthesised samples was carried out using a Bruker DPX-300 NMR (300 MHz) nuclear magnetic resonance spectrometer (Billerica, MA, USA). Samples were dissolved in deuterated chloroform (10 mg of samples in 1 mL of solvent), and tetramethyl silane was used as the referent standard.

The thermal properties of the samples were evaluated using differential scanning calorimetry (DSC) (TA Instruments Q20, New Castle, DE, USA). Approximately 5 mg of each sample was placed in hermetically sealed aluminium pans. The samples were heated from 20 to 180 °C at a rate of 10 °C min^−1^, cooled to −90 °C at 5 °C min^−1^, and then subjected to a second heating to 270 °C at 10 °C min^−1^. The nitrogen flow rate was 50 mL min^−1^. Indium was used as the reference for calibration of the equipment’s temperature.

Thermogravimetric analysis (TGA) was carried out using a TA Instruments Q500 instrument. Samples of around 10 mg were heated from 30 °C to 600 °C, with a heating rate of 10 °C min^−1^ under a nitrogen atmosphere with a gas flow rate of 60 cm^3^ min^−1^. Weight loss and temperature difference were recorded as a function of temperature.

Wide-angle X-ray scattering measurements were taken at room temperature using a SAXSess mc^2^ instrument (Anton Paar, Graz, Austria) with monochromatised CuKα radiation (λ = 0.1542 nm) generated by a GeniX microfocus X-ray source (Xenocs SA) at 50 W power. The X-ray detector used was a PerkinElmer Cyclone^®^ Plus Storage Phosphor System (Waltham, MA, USA). The measurements were performed in transmission mode. The degree of crystallinity was calculated as the ratio of the crystallinity peak area to the total peak area in the 2θ range of 5–25°.

## 3. Results and Discussion

Table 1 displays the results of the average molecular weight analysis. Average molecular weights (*Mn*) for PU samples were in the range from 55,000 to 61,000 g mol^−1^. The samples with PCL as the polyol component had lower polydispersity indexes compared with samples with PCL/PDLLA polyol, which is expected due to the different reactivity of the two polyol components. However, the different reactivity of the polyols had no influence on the values of the *Mn*, which is explained by the sufficient reactivity of both polyols to form polyurethane. 

The primary FTIR bands and their respective functional groups in the polyurethanes are shown in Table 2. The FT-IR spectra of the PU sample containing PCL and PDLLA as polyol components exhibit characteristic bands associated with urethane stretching (N–H) at 3459 and 3539 cm^−1^, a urethane carbonyl (NH–CO–O) at 1710 cm^−1^ (which is overlapped with C=O carbonyl of PCL ester group), and a combination of N–H out-of-plane bending and C–N stretching at 1542 cm^−1^. The presence of bands at 3539 and at 1750 cm^−1^ indicate that NCO had reacted with OH groups, converting them to HN–CO–O. Furthermore, it can be observed that all the NCO groups of the diisocyanate had undergone reaction, as the band corresponding to unreacted NCO groups is not detected at 2250 cm^−1^. 

**Figure 2:**
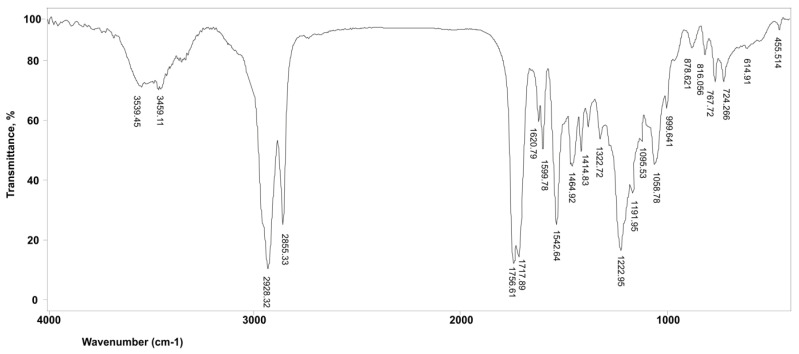
FTIR spectrum of synthesised PU sample, based on polycaprolactone diol (PCL) and poly(D,L-lactide) (PDLLA) as polyol components.

In the FT-IR spectra of the samples with PDLLA as their polyol component, notable shifts can be observed in the asymmetrical valence vibrations of C–O–C (1191 cm^−1^) and symmetrical valence vibrations of C–O–C (1095 cm^−1^) of the aliphatic chain in the poly(D,L-lactide) block compared with bands at 1267 and 1099 cm^−1^, respectively, present in monomer L-lactide [12]. Additionally, the presence of bands at 1464 cm^−1^ (δOH) and 767 cm^−1^ (γOH) indicates terminated hydroxyl groups in the PDLLA and PCL chains, thus confirming our presumption regardingthe above-explained polymerisation mechanism. The peak which corresponds to the vibration of C=O group from ester polyols is split into two bands originating from the PDLLA C=O at 1756 cm^−1^ and PCL C=O at 1717 cm^−1^, as shown in Figure 2. 

**Figure 3:**
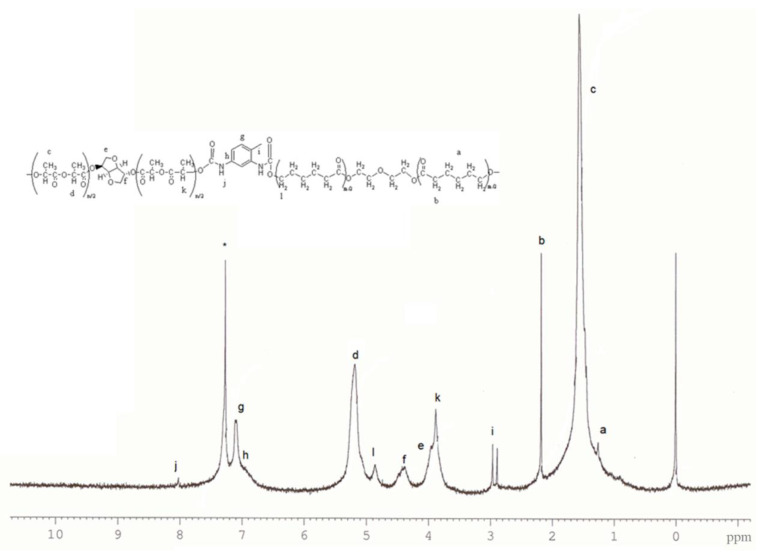
^1^H NMR spectrum of synthesised polyurethane (sample PU4).

^1^H NMR spectroscopy was used to confirm the structures P of synthesised PU samples. Proton signals at 1.21 (a) and 2.2 (b) represent central methylene protons from the PCL-polyol chain. Peaks present at 1.6 ppm (c) and 5.2 ppm (d) of poly(D,L-lactide) synthesised with isosorbide can be attributed to methyl and methine proton resonances of the stereosequence, originating from lactic acid in the polyester chain, as shown in Figure 3. The peak at 4.5 ppm (f) corresponds to the proton originating from isosorbide, which is located in the middle of the PDLLA chain. Additionally, the peak at 3.8 ppm (e) originates from the protons from methylene groups in the isosorbide ring, –O–C**H**_2_–CH–O–. At 7.17 ppm (g) and a small left shoulder (h) originate from the protone in benzene ring. The methyl group from TDI is confirmed by the presence of the peak at 2.98 ppm (i). Confirmation of urethane bond formation, i.e., the reaction of isocyanate –NCO and polyol –OH groups, is evidenced by the appearance of new peaks: one at 8.05 ppm (j) from the NH protone of the newly formed urethane bond and another methine proton from the PDLLA chain near the urethane bond, which, due to the change to their surroundings, shifted to 3.9 ppm (k). Formation of urethane bond with PCL diol also changed the position of NH–COO–C**H_2_**– shifting it to 4.85 ppm (l). These new peaks confirm the reaction of TDI with both polyol components and urethane formation. The peak at 7.26 ppm is attributed to the solvent CDCl_3_ and is denoted by an asterisk (*).

### 3.1. Wide-Angle X-ray Scattering

**Figure 4:**
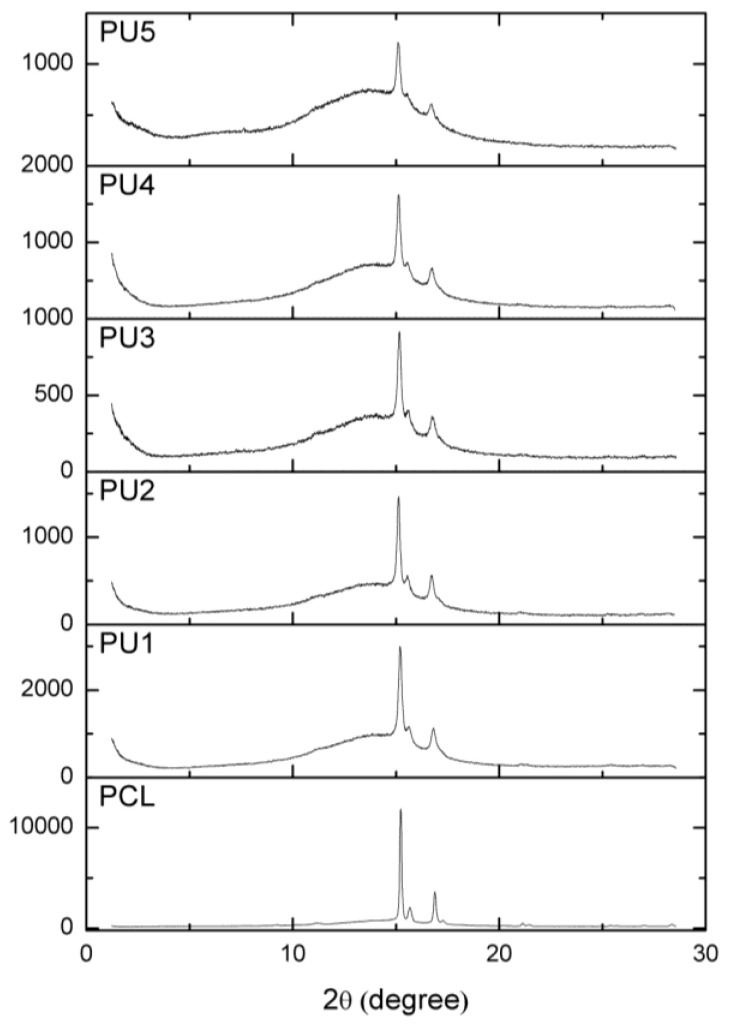
WAXS spectra of PU samples and pure PCL.

Wide-angle X-ray scattering (WAXS) was employed to examine the crystalline phase of the PUs. The samples were cast from a solution and left to dry at room temperature for two days. The WAXS spectra (Figure 4) confirm that all the prepared materials demonstrate semicrystalline behaviour, as evidenced by the presence of several scattering peaks attributed to crystallites of PCL.

The degree of crystallinity was calculated by the deconvolution method using a Gaussian–Lorentzian sum (MagicPlot 3.0 software). The positions of the scattering peaks observed in the PUs align well with the peaks observed in pure PCL (at 15.2, 15.6 and 16.8°). This finding indicates that the crystalline phase in the synthesised PU is associated with the crystallisation of the PCL soft segment, as the PDLLA remains amorphous. The impact of the PCL/PDLLA polyol ratio on the crystallisation ability of the PCL is clearly evident. 

The values of the degree of crystallinity, χ_c_, obtained from WAXS spectra are presented in Table 3. The sample with pure PCL as the polyol component (PU1) exhibits the highest degree of crystallinity (34.85%).

The degree of crystallinity of solution-cast PU samples, as deduced from the WAXS spectra, exhibits a decreasing trend in the following sequence: PU1 > PU2 > PU3 > PU4 > PU5, namely with increasing of the portion of the amorphous PDLLA polyol.

As the PDLLA soft segment content increases from 0.2 to 0.8, there is no hindrance of the degree of connectivity between the hard and soft segments, which indicates that the combination of selected polyester soft segments yields quantitative reactions between soft and hard segments, producing polyurethanes. The observed decrease in χ_c_ as the PDLLA content increases indicates that the crystallites formed during the crystallisation from the solution of the PCL soft segment in the PU are not as perfect as those developed in the initial PCL diol. The degree of crystallinity values obtained from WAXS is lower than that of the values obtained from the DSC results. This difference arises because χ_c_ calculated using WAXS is based on the ratio between the scattering of the crystalline PCL phase and the overall X-ray scattering of the sample, including the crystalline PCL diffraction and the amorphous regions of PDLLA and hard segments. On the other hand, in DSC analysis, αc represents the crystallinity of the PCL phase alone.

### 3.2. DSC Study

**Figure 5:**
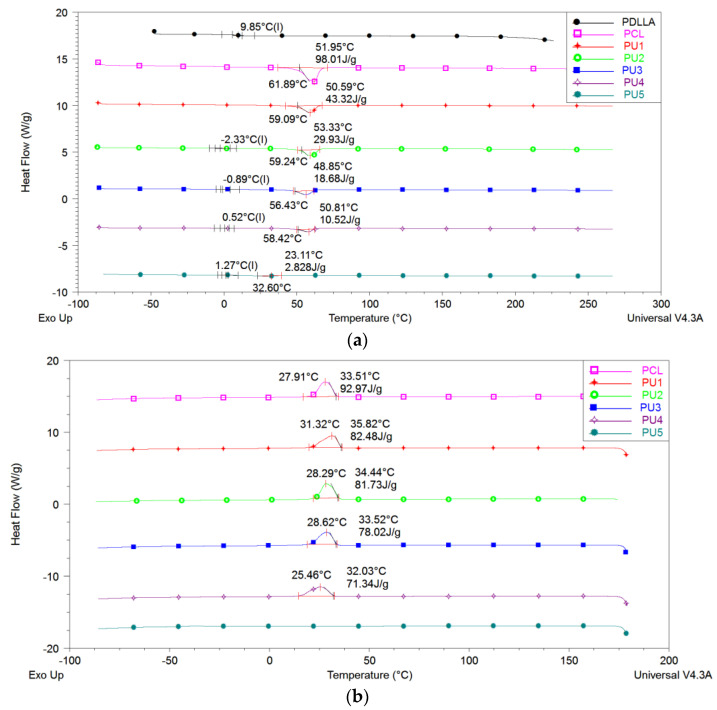
DCS thermograms of PU samples: (**a**) 2nd heating (10 °C min^−1^), (**b**) cooling (5 °C min^−1^).

Investigation of the polyurethane series demonstrated the influence of the PCL soft segment on the crystallinity increase of the materials, unlike conventional PU materials containing crystalline hard microphase. To validate this, DSC results of the pure PCL diol were compared with those of PU samples containing polyol components. Figure 5a represents the second heating scans, revealing lower melting temperatures (T_m_) and melting enthalpies (Δ*H*_m_) in PU samples compared to neat PCL diol, which is consistent with the anticipated results.

In PU1, the sample containing only PCL polyol as the soft segment, the crystalline domains exhibit the highest melting temperature (59 °C) and the highest melting enthalpy (43.32 J g^−1^). The decrease of the heat of fusion is observed upon incorporating PDLLA polyester polyol into the polyurethane structure (samples PU2 to PU5). The presence of amorphous PDLLA as the soft segment hinders the crystallisation of the PCL soft segment in PUs. Notably, in PU5, which contains 80 wt% of PDLLA, a small endothermic peak appears at much lower temperature compared to pure PCL, indicating the possibility of PCL crystallisation in this PU sample. 

The decrease in the crystallinity of the PCL soft phase in PUs compared to the neat PCL diol indicates that the presence of amorphous PDLLA segments strongly hinders the crystallisation of PCL soft segments in the copolymers. The degree of crystallinity, α_c_, of the PCL phase was determined using a neat crystalline PCL Δ*H*_m_ (139.5 J g^−1^) [26]. The results confirm our finding that the crystals formed during the crystallisation process from the solution of the PCL soft segment in the PU chain have more irregularities compared to those formed in the neat PCL polyol, as previously observed via WAXS analysis (Table 4).

The first heating scans were conducted on the pristine samples immediately after the synthesis process and solvent film casting in order to erase the thermal history of the samples. All the following cooling scans (ranging from 180 to −90 °C at 5 °C min^−1^ (Figure 5b)) revealed a crystallisation at temperatures ranging from 25 to 31 °C, which is nearly the same as the crystallisation temperature observed for neat PCL diol (~28 °C). However, the value of observed crystallisation enthalpy decreased with an increase of PDLLA content in the polyol mixture. The sample with the highest PDLLA content, PU5, has not shown crystallisation cooling regime, while in the second heating, only a small, 2.83 J g^−1^, endos peak appears. 

After analysing the DSC cooling thermograms, it was once again observed that amorphous PDLLA polyol inhibits the crystallisation of PCL during cooling, as shown in Table 4. Additionally, the interaction between the hard and soft segments in the polyurethane plays a role in reducing the flexibility of the chain. This interaction has the additional effect of hindering the crystallisation process of the PCL segments, contributing to the overall suppression of PCL crystallisation.

### 3.3. TGA Analysis

The TGA curves of the PU samples offer insights into the thermal degradation processes, which are influenced by the chemical structures of the soft and hard segments. The degradation mechanism of polyurethane is intricate, resulting in the formation of various products, reflected in multiple peaks in the derivative curves. According to the literature [27,28], the thermal degradation of polyurethanes is initiated at approximately 230 °C, corresponding to the degradation of the hard segments formed by urethane and urea bonds. The degradation of the more stable soft segments occurs at higher temperatures.

**Figure 6:**
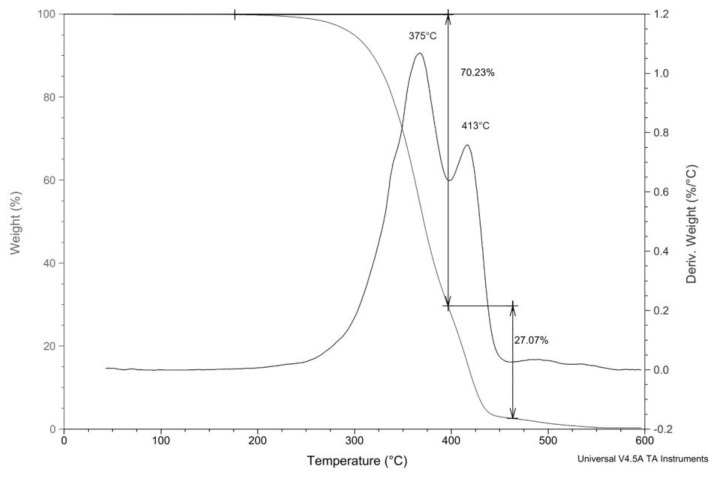
TGA and DTG thermograms of the polycaprolactone diol.

To investigate the influence of soft segment polyols, pure PCL and PDLLA were analysed using TGA. The TGA thermogram of the PCL diol shows primary degradation at 375 °C, followed by a weaker process at around 337 °C and a stronger one at 413 °C (Figure 6). 

**Figure 7:**
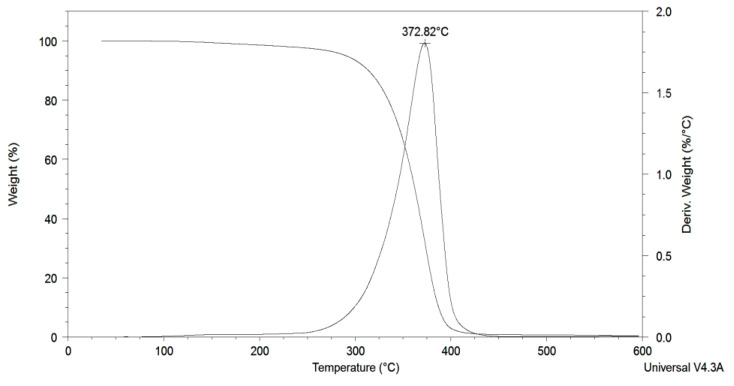
TGA and DTG thermograms of the poly(D,L-lactide) diol.

Figure 7 presents the thermal degradation profile of synthesised poly(D,L-lactide) polyol. The low thermal stability and a degradation onset temperature at 323 °C are explained by high stereoregularity because of cationic polymerisation. Based on the proposed cationic lactide polymerisation mechanism, –OH and, to a lesser extent, C(O)OSO_2_CF_3_ end-groups both endow the growing macromolecules. According to Jamshidi [29], the presence of non-polymeric contents in the reaction medium enhances the thermal stability of the resulting polymers and facilitates the blocking of hydroxyl end-groups during polylactide synthesis. However, the inclusion of isosorbide in the middle of polymer chains does not significantly impact the thermal stability of their copolymers, as thermal degradation primarily initiates from the chain ends [9]. 

**Figure 8:**
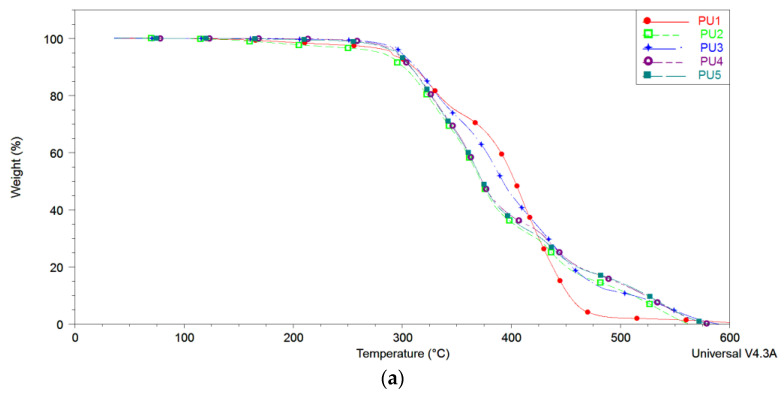

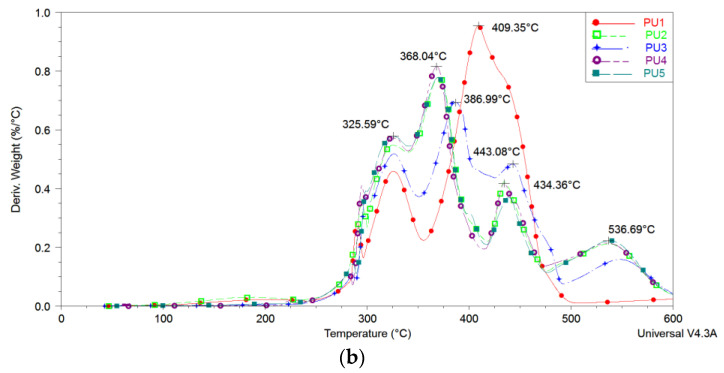
(**a**) TGA (**a**,**b**) DTG thermograms obtained from polyurethanes with varying soft segment contents.

To gain a deeper understanding of the decomposition processes, the DTG curves of all synthesised polyurethane materials were analysed, as depicted in Figure 8. The derivative curves of the presented polyurethanes reveal distinct degradation stages: around 325 °C, indicating the initiation of polyurethane bond degradation; at 370 °C, signifying the degradation of soft segments; at 430 °C, indicating the degradation of the hard segments; and above 500 °C, representing the degradation of the residue (Figure 8). Additionally, between 110 and 250 °C, the removal of hydrogen-bonded water and residual solvent is observed. The composition of soft segments and the molecular weight of the polyol lead to shifts in PU peaks due to the degradation of both hard and soft segments. The thermal stability of polyurethanes depends on the chemical composition and molecular weight distribution of the polyol incorporated as soft segments into the polymer backbone [30,31]. Coutinho et al. [32] concluded that longer soft segment lengths result in increased thermal stability in polyurethanes. However, based on the TGA results presented in Figure 7, the impact of soft segment structure and chain length of polyether and polyester polyols on the thermal stability of polyurethanes is not significant. This finding is very important in terms of the application of polyester-based PU, especially for high-temperature applications.

It is interesting to note that the PU sample with only PCL as a soft segment practically shows two main peaks of decomposition: at 325 °C, which marks the beginning of the decomposition of the polyurethane bonds in the main chain, and at 409 °C, which corresponds to the simultaneous decomposition of soft and hard segments, as confirmed by the appearance of a “shoulder” at around 440 °C. This peak marks the decomposition of hard segments, which is more clearly visible in PU samples with the addition of PDLLA, due to a more pronounced microphase separation of hard and soft blocks. Considering that the decomposition temperatures of the soft segments (PCL and PDLLA) are almost identical, at 370 °C, in samples containing both polyols, the decomposition of the soft segment takes place in one step at temperatures of about 368 °C, Figure 7. Due to the similar decomposition temperatures observed in samples with the addition of PDLLA soft segments, determining the specific influence of PDLLA polyol on the thermal stability of the prepared materials becomes challenging. In summary, it is estimated that the sample PU3 with 60/40 *w*/*w* PCL/PDLLA ratio shows a shift of the peaks at the higher temperature, because the polyether PCL block enables higher thermal stability, enhanced by the phase separation of hard and soft segments enabled by the presence of PDLLA block. Other samples, namely PU2, PU4 and PU5, showed almost identical thermal degradation profiles, with degradation of the soft segments occurring at 325 °C and 368 °C and degradation of the hard segments at 434 °C.

The onset degradation temperature of all synthesised PU samples is identical (Table 4). This temperature corresponds to the degradation of polyurethane bonds, indicating that the type and proportion of polyols used had no influence on the thermal stability of the PU samples. The first degradation step, which marks the initiation of PU degradation by breaking polyurethane bonds, also occurs at the same temperature for all samples. A fourth degradation step, observed only in the samples with PDLLA polyols, shows residue, confirming the influence of polyester polyol on the final step of the degradation mechanism. 

Table 5 shows that in sample PU5, with its high PDLLA content in the soft segment, two peaks were observed during the decomposition of the soft segment (369 °C and 401 °C). The presence of the second peak can be explained by the second stage of decomposition of the PCL block (Figure 5), which becomes more pronounced with an increase in the ratio of the polyester segment. This leads to separation in the soft segment itself and possibly a different mechanism of their incorporation in the presence of a large excess of the polyester segment. Additionally, varying the type and composition of polyester polyols did not influence the thermal decomposition of the hard segments, as expected.

## 4. Conclusions

Novel polyurethanes based on polyol components were successfully synthesised without the use of a chain extender via a one-step bulk polymerisation process. PUs were prepared using aromatic diisocyanate, TDI, and differing ratios of PCL and synthesised PDLLA as polyols. Varying the molar ratio of newly synthesised PDLLA in soft segments played a crucial role in fine-tuning the final properties of the PU. The FTIR experiments have shown that the polyol type had no influence on the PU chemistry and that novel PDLLA polyol exhibited the desired reactivity for PU synthesis. The introduction of PDLLA polyol in the soft segment significantly impacted the degree of microphase separation in both the soft and hard segments, indicating a clear connection to the crystallisation behaviour of the resulting polyurethane. It was observed that all polyurethane samples containing PCL as part of the soft segment exhibited crystallisable properties. However, the degree of crystallinity decreased as the PDLLA content increased (from 34.8% in the sample with 0 wt% of PDLLA in the soft segment to 12.4% in the sample with 80 wt% of PDLLA in soft segment), suggesting that amorphous PDLLA hindered PU crystallisation. Crystallisations of PCL/PDLLA polyols in their soft segment composition. DSC data obtained from the second heating run revealed that all PU samples with different ratios of polyol components exhibited a crystalline structure originating from PCL. However, the DSC cooling thermograms indicated that the presence of amorphous PDLLA polyol not only inhibited PCL crystallisation during cooling but also influenced the interaction between the hard and soft segments, reducing chain flexibility and hindering PCL crystallisation. Values of crystallisation enthalpy decreased with increases to the PDLLA content in the polyol mixture. The sample with the highest PDLLA content did not show crystallisation during the cooling regime, while during the second heating, only one small, 2.83 J g^−1^, endo peak appears. The onset degradation temperature for all synthesised PU samples is identical, indicating that the type of polyols used and their proportion had no influence on the thermal stability of PU samples. Varying the type and composition of polyester polyols did not influence the thermal decomposition of hard segments, as expected. It can be concluded that the addition of a novel synthesised poly(D,L-lactide) polyol to the polyurethane formulation offers a promising approach to achieve the desired performance of biocompatible polyurethanes. These findings open up new possibilities for the development of advanced biomaterials for various applications.

## Figures and Tables

**Table 1:** The compositions and average molecular mass (*Mn* and *Mw*) and polydispersity index of synthesised polyurethane.

Sample	PCL (mol)	PDLLA (mol)	TDI (mol)	*Mn*/g mol^−1^	*Mw*/g mol^−1^	*Q*
PU1	1	/	1.2	55,874	84,928	1.52
PU2	0.80	0.20	1.2	60,987	112,216	1.84
PU3	0.60	0.40	1.2	59,348	139,467	2.35
PU4	0.40	0.60	1.2	61,545	112,011	1.82
PU5	0.20	0.80	2	59,986	118,772	1.98

**Table 2:** The primary FTIR bands and their respective functional groups in the polyurethanes derived from PCL, PDLLA, and toluene diisocyanate.

Polymer Frequency (cm^−1^)	Assignment	Remark
3539–3459	ν (N–H)	N–H stretching of urethane bond, free and hydrogen bonded NH
2928–2855	ν (CH_2_)	Asymmetric 2925 cm^−1^Symmetric 2855 cm^−1^(with additional weak shoulder around 2960 cm^−1^ ν (–CH_3_) reflecting the presence of methyl groups in the polyol components)
1756	ν (C=O)	Valence vibration of C=O of aliphatic poly(D,L-lactide) chain
1717	ν (C=O)	Valence vibration of C=O of aliphatic poly(caprolactone) chain
1710	ν (HNCOO)	Stretching frequency of the urethane carbonyl groups
1542	Amide II	N-H bend (amide II)
1191–1095	ν (C–O–C)	Asymmetric 1191 cm^−1^ and symmetric 1058 cm^−1^ vibration of ester bond in PDLLA and PCL
1599	ν (C=C)_aromatic_	Vibration of the C=C double bond in the aromatic rings
880–724	γ(C–H)	Out–of–plane C–H

**Table 3:** The degree of crystallinity of the polyurethane samples determined through WAXS analysis.

Sample	PCL/PDLLA (mol/mol)	χ_c_ (%)
PU1	1/0	34.8
PU2	0.8/0.2	28.5
PU3	0.6/0.4	26.6
PU4	0.4/0.6	17.9
PU5	0.2/0.8	12.4
pure PCL	-	54.3

**Table 4:** Thermal properties of the polyurethanes and the PDLLA and PCL diols determined from DSC.

Sample	2nd Heating (10 °C min^−1^)				Cooling (5 °C min^−1^)	
	*T*_mSS_ (°C)	Δ*H*_mSS_ (J g^−1^)	α_c_ (%)	*T_gHS_* (°C)	*T_c_* (°C)	Δ*H_c_* (J g^−1^)
PCL	61.89	98.01	70	/	/	/
PDLLA	/	/	/	9.85	/	/
PU1	59.09	43.32	41	/	27.91	92.97
PU2	59.24	29.93	36	−2.33	31.32	82.48
PU3	56.43	18.68	30	−0.89	28.29	81.73
PU4	58.42	10.52	24	0.52	28.62	78.02
PU5	32.60	2.83	14	1.27	25.46	71.34

**Table 5:** Decomposition temperatures of the polyurethanes with various soft segment structures, determined from the TGA analysis.

Sample	Onset Temperature	1st Decomposition Step	2nd Decomposition Step (Soft Segment)	3rd Decomposition Step (Hard Segment)	4th Decomposition Step (Residue)
	T (°C)	T (°C)	T (°C)	T (°C)	T (°C)
PU1	295.62	325	409	439	/
PU2	290.80	325	370	434	537
PU3	295.78	326	386	443	548
PU4	288.11	325	368	434	534
PU5	291.97	325	369, 401	438	536

## Data Availability

Not applicable.
